# How the Degree of Anthropomorphism of Human-like Robots Affects Users’ Perceptual and Emotional Processing: Evidence from an EEG Study

**DOI:** 10.3390/s24154809

**Published:** 2024-07-24

**Authors:** Jinchun Wu, Xiaoxi Du, Yixuan Liu, Wenzhe Tang, Chengqi Xue

**Affiliations:** School of Mechanical Engineering, Southeast University, Suyuan Avenue 79, Nanjing 211189, China; wjcseu@seu.edu.cn (J.W.); duxiaoxi@seu.edu.cn (X.D.); 230229259@seu.edu.cn (Y.L.); wenzhe.tang@seu.edu.cn (W.T.)

**Keywords:** anthropomorphism, anthropomorphic robots, emotional responses, perception, ERPs, ERSP

## Abstract

Anthropomorphized robots are increasingly integrated into human social life, playing vital roles across various fields. This study aimed to elucidate the neural dynamics underlying users’ perceptual and emotional responses to robots with varying levels of anthropomorphism. We investigated event-related potentials (ERPs) and event-related spectral perturbations (ERSPs) elicited while participants viewed, perceived, and rated the affection of robots with low (L-AR), medium (M-AR), and high (H-AR) levels of anthropomorphism. EEG data were recorded from 42 participants. Results revealed that H-AR induced a more negative N1 and increased frontal theta power, but decreased P2 in early time windows. Conversely, M-AR and L-AR elicited larger P2 compared to H-AR. In later time windows, M-AR generated greater late positive potential (LPP) and enhanced parietal-occipital theta oscillations than H-AR and L-AR. These findings suggest distinct neural processing phases: early feature detection and selective attention allocation, followed by later affective appraisal. Early detection of facial form and animacy, with P2 reflecting higher-order visual processing, appeared to correlate with anthropomorphism levels. This research advances the understanding of emotional processing in anthropomorphic robot design and provides valuable insights for robot designers and manufacturers regarding emotional and feature design, evaluation, and promotion of anthropomorphic robots.

## 1. Introduction

Robots are increasingly being used in a variety of human social life situations and play a vital role in numerous fields, including industrial production, healthcare, homecare, education, and entertainment [1,2,3]. Given the diverse range of backgrounds, training, physical capabilities, and cognitive skills among users in the field of Human-Robot Interaction (HRI), it is expected that robots should possess qualities such as intuitiveness, ease of use, trustworthiness, high user acceptance, and responsiveness to meet the demands and states of their users. To facilitate robot service and overcome challenges (e.g., trust and acceptance of robots) to HRI, robot researchers and engineers have designed anthropomorphic (resemble humans or human-like design) robots [4,5,6]. The attribution of humanlike characteristics to nonhuman or inanimate entities is referred to as anthropomorphism and has been widely adopted as a supporting design feature in the robotics field [7]. Positive reactions to anthropomorphic robots often include increased trust, empathy, and cooperation. For instance, healthcare robots designed with human-like features have been shown to improve patient comfort and compliance with medical instructions [8]. Similarly, in educational settings, anthropomorphic robots can foster a more engaging and interactive learning environment, leading to improved educational outcomes [9]. However, not all reactions to anthropomorphic robots are positive. Negative responses can arise due to the uncanny valley effect, where robots that appear almost human, but not quite, elicit feelings of eeriness and discomfort. An example of this is seen in customer service scenarios, where overly human-like robots may cause unease and decrease customer satisfaction [10]. Additionally, there are concerns about privacy and security, as anthropomorphic robots equipped with advanced sensors and data processing capabilities may be perceived as intrusive [11]. Thus, it is not always the case that the more anthropomorphic a robot is, the better it is perceived to be [12]. It is believed that anthropomorphism, when used properly, can significantly alter users’ attitudes and improve robot marketing [13]. Hence, it is of great importance to investigate how the anthropomorphism of robots affects humans’ perceptions, emotional responses, attitudes, and evaluations.

A number of studies have been conducted exploring factors influencing how users perceive, judge and evaluate anthropomorphic robots, mainly from aspects of the uncanny valley effect [14,15], robot appearance [13,16,17,18,19], facial expressions of emotion [20,21], robot actions [22,23,24], human-robot interaction [25,26,27], even gender [28], personality [29], and robot anxiety [30]. However, most studies aforementioned have tended to use the traditional approaches based on questionnaire surveys and interviews, which may lead to subjective bias and make it difficult to obtain individuals’ true perceptual and cognitive information [13,14,31,32]. Recently, several studies have attempted to investigate users’ preferences, robot actions and emotional behaviors of humanoid robots using quantitative psychological monitoring techniques, such as functional magnetic resonance imaging (fMRI) [33], eye tracking (ET) [31,34], electroencephalography (EEG) [23,35], and event-related potentials (ERPs) [23,31,33,36,37,38,39]. While these efforts are innovative and meaningful for anthropomorphic robot research, they tend to focus on responses to particular features (e.g., appearance or actions) of the same categorical robots (e.g., humanoid robots). Few have explored how the degree of anthropomorphism of human-like robots affects users’ perceptual processing and emotional responses from the time dimension of neuroprocessing, and there was a lack of studies regarding robotic anthropomorphism that looked at the brain areas associated with emotional processing [40].

**Figure 1:**
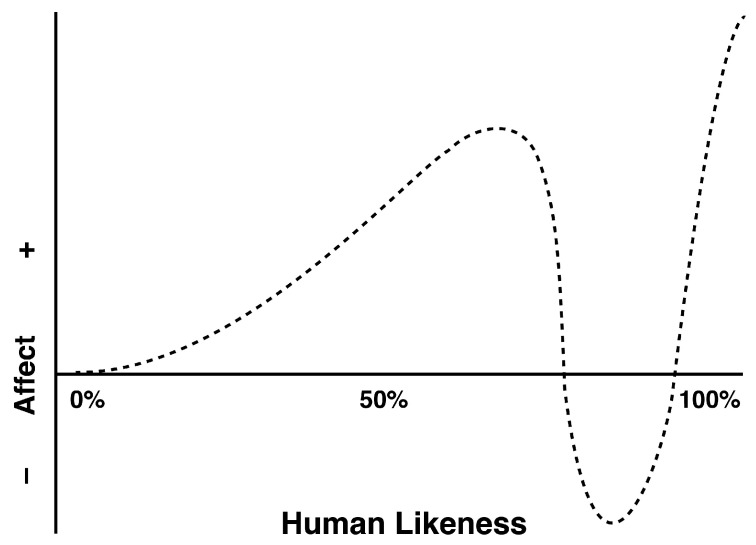
The uncanny valley function, as proposed by Mori (1970) [10].

Evaluating users’ perceptual processing, emotional processing and attitudes toward anthropomorphic robots could provide guidelines for potential improvements in the design of emotionally anthropomorphic robots. It is, therefore, important for anthropomorphic robot designers to ensure that the robot can accurately elicit users’ positive emotional responses. While Mori’s theory [10] depicted the relationship between human-likeness and affective reactions (see Figure 1), the emotional valence and arousal related to the degree of anthropomorphism of human-like robots and the corresponding underlying neural dynamics have not been fully considered. Some studies have sought to investigate the impact of the uncanny valley effect on affective responses and psychophysiological reactions. For instance, Cheetham et al. [36] examined affective experience in response to categorically ambiguous compared with unambiguous avatars and human faces by using EEG, electromyography (EMG) and self-report indices. The LPP and SAM-based measures of arousal and valence indicated a general rise in negative affective state (i.e., enhanced arousal and negative valence) with greater morph distance from the human end of the dimension of human likeness. However, the stimuli used in the study were drawn from controlled morph continua of human faces. This way of controlling anthropomorphism via the face dimension (changing outward surface features of a face) may not be applicable to real-life robots, which are more diverse and have varying degrees of anthropomorphism (e.g., middle anthropomorphic robots, toy robots, service robots, etc.).

Accordingly, the present study aimed to investigate the time course of neural dynamics involved in the users’ perceptual and emotional processing in response to robots with different levels of anthropomorphism, to evaluate the relationship between users’ subjective emotional valence, arousal, and electrophysiological data, as well as assess user’s attitudes toward anthropomorphic robots, such as perceived likeability and warmth. In this study, we combined the subjective evaluation and the objective electrophysiological measurements. Forty-two participants were equipped with an EEG device to record the electroencephalogram (EEG) data while performing an affective rating task, and ERPs and ERSP were analyzed to reveal their information processing stream while viewing, perceiving and evaluating the appearance of anthropomorphic robots. The subjective ratings were utilized to complement the objective data. Attitudes toward anthropomorphic robots were gleaned by assessing individuals’ perceived likeability and warmth. The results might advance our understanding of how the anthropomorphism of an anthropomorphic robot affects users’ perceptual or cognitive processes and emotional responses from the underlying neural level. Moreover, the findings might contribute to a better understanding of the cognitive underpinnings of the uncanny effect. In practice, the findings could provide reference and guidelines for emotional design, feature design, assessment, marketing, implementation, and promotion of anthropomorphic robots for robotics designers and manufacturers.

## 2. Related Work

### 2.1. Anthropomorphism of Robots

Anthropomorphism is the attribution of external humanlike characteristics such as the face, nose and mouth, and internal characteristics such as motivations, intentions and emotions to nonhuman or inanimate entities [7,41]. When humans see a schema consistent with their owns in a nonhuman object, it will be regarded that the nonhuman object is anthropomorphized [41]. As a new marketing method, anthropomorphism is often applied in robot design [42,43], product design [44,45,46], icon design [47] and brand promotion [48,49,50]. Anthropomorphic robots realize anthropomorphism by mimicking the physiological and psychological states of humans [21,51], as well as human-like personality traits [30,52]. Robots’ actions, emotional facial expressions, voice, and efficiency of human-robot interaction all have an influence on users’ perceptions and evaluations regarding anthropomorphism [21,41,53].

Regarding the measurement of robots’ anthropomorphism, most academics have concentrated primarily on traditional methods such as subjective ratings. MacDorman [54] proposed a questionnaire with a single question to assess human-likeness. Powers and Kiesler [18], in comparison, suggested a different anthropomorphic questionnaire that contained six items and appeared to have a higher level of reliability. Bartneck et al. [42] developed a consistent questionnaire for detecting anthropomorphism using semantic differential scales based on prior research. Roesler et al. [55] used the frequency of choosing robots and the response latency of every selection to measure the preferred degree of anthropomorphism. Subjective measurement of anthropomorphism has its own advantages, but it has been limited by the subjective bias of individuals and may introduce psychometric noise into the self-reported ratings [56]. Thus, devising an objective means to measure the anthropomorphism of human-like replicas is crucial. Some studies have explored the unconscious behavior of users by employing covert anthropomorphic measurements. For instance, Minato et al. [57] examined participants’ gaze patterns while gazing at either a human or an android. In a follow-up study, Shimada et al. [34] examined the similarity between the gaze behavior (i.e., duration and gaze orientation) toward an android robot and a human, and found that an android with a higher physical resemblance to a human evoked a more human-oriented gaze, suggesting that gaze behavior can be utilized to implicitly quantify robot anthropomorphism. Moreover, in other studies, anthropomorphism has been manipulated and measured objectively by altering the outward surface features of a face, converting a real human face into a synthetic one, or manipulating robot personality traits [30,58,59,60].

Although many studies have been conducted to evaluate and measure the anthropomorphism of robots and demonstrated that robots can be anthropomorphized through anthropomorphic appearances and behaviors, few have investigated the neural mechanisms behind how the degree of anthropomorphism of human-like robots affects individuals’ perceptual and emotional processing. Uncovering the neural dynamics underlying users’ perceptions and affective processing of robots varied in their degree of anthropomorphism may help design emotionally anthropomorphic robots.

### 2.2. Emotional Responses, Likeability, and Warmth on Robots

A growing body of studies has shown that the anthropomorphism of robots was responsible for people’s emotional responses, likeability and warmth [13,14,19,58]. Concerning emotional responses, Mori’s uncanny valley theory [10] holds significant importance and should not be disregarded. It elucidates the relationship between robots’ human likeness and humans’ emotional responses. While Mori’s theory showed how affective responses vary with the perceived human likeness, emotional valence and arousal have not been fully considered, particularly the valence dimension [10]. Additionally, most research has generally relied on the conventional methods of emotional evaluation based on questionnaire surveys, which may introduce subjectivity bias or memory bias, and provide difficulties or constraints in eliciting true information about individuals’ perceptual and affective processing. Thus, it is crucial to develop an objective way to quantify emotional processing while evaluating robots with different degrees of anthropomorphism. Furthermore, previous research has demonstrated that emotional valence is positively related to emotional experience, while higher emotional arousal corresponds with good or poor affective experience and lower arousal with a moderate emotional experience [61,62]. Based on the uncanny valley effect, robots with a high level of anthropomorphism often have highly anthropomorphized faces, which may give people a potentially weird, uncomfortable feeling of uncanniness and evoke a poor emotional experience. Robots with a low level of anthropomorphism exhibit more adorable non-human characteristics that may induce a good affective experience. 

In terms of likeability, it has been argued that early favorable impressions (e.g., likeability or affinity) often result in more positive assessments of an individual [14,42,63]. It has been shown that people are prone to make judgments and form attitudes in the instants immediately after encountering another person. Since robots are regarded as social actors to some extent, it can be assumed that people are capable of judging robots in a similar manner [64,65]. In the present study, the degree to which users have favorable perceptions and evaluations of robot looks was referred to as the users’ likeability for robots. According to Mori’s hypothetical curve, to a point, likeability increased with increasing human-resemblance, but as robots became more human-like (such as robots with high level of anthropomorphism), they began to be perceived as very unlikeable. 

With regard to human warmth, it has been associated with social attributes such as caring, nice, and sociable traits, as well as friendliness, and trustworthiness [13,66]. It has been revealed that warmth judgments are more relevant than competence in affective and behavioral responses, despite the fact that their emergence as dimensions of warmth and competence was consistent [13,41,67]. People’s positive attitudes have been found to be closely linked to an individual’s level of warmth; thus, the warmer an individual is, the more positive people’s attitudes are as well [68]. Similar findings have been reported in late anthropomorphic brand research [48], as well as virtual agents [65]. The Uncanny Valley theory [10], suggests that the affinity fades when robots become too human-like, and people will experience an uncomfortable feeling of uncanniness. Thus, it can be assumed that when people anthropomorphize robots and perceive them as growingly warm in external appearance, the increase in warmth will be perceived positively at first. However, once robots resemble humans too closely, an uncomfortable feeling of uncanniness should creep in and result in a negative shift in attitude. 

### 2.3. Electrophysiological Components Related to Perceptual Processing, Affective Processing, and Evaluation

The ERP technique is a useful tool in the investigation of unveiling physiological aspects that affect users’ behavior and preferences, and it can also help investigate the “common scale” that makes it possible to compare users’ heterogeneous and individualized behaviors [69]. Numerous studies have explored the neural dynamics involved in the processing of affective information. However, most prior ERP studies on affection have typically utilized emotional pictures as stimuli from IPAS [70], and some have also used other stimuli, such as arts [71], faces [72], humidifiers [73], webpages [62], and even logos [74]. Results from these studies have identified P1, N1, P2, N2, P3, and LPP components as sensitive to affective contents and affective processing of the stimuli. Two affective ERP components have often been the focus of emotional studies: one is the early posterior negativity (EPN), a negative potential that is primarily distributed across the visual cortex in the time window of 230–280 ms; the other is the late positive potential (LPP), a positive potential that typically held long-lasting increased positivity, and exhibited a comparable onset time and activated cortical distribution as P3. In the current study, the ERP components of interest were exogenous N1, P2, and endogenous LPP. 

#### 2.3.1. The N1 Component

The visual N1, which has a wide scalp distribution and peaks earlier in the frontal than in the posterior regions [75], is closely related to the early visual processing of emotional stimuli and affective pictures. For instance, Keil et al. [76] revealed that strengthened N1 can be elicited by both positive and negative stimuli relative to neutral stimuli. It has been identified that the N1 component could index the distribution of attentional resources in response to stimuli and might have an effect on perceptual processing, such as the selection and discrimination of perceptual features (i.e., color, appearance and shape) [73,77,78]. Guo et al. [37] suggested that the preferred humanoid robot appearances elicited a larger N1 amplitude. Convincing evidence also has indicated that the N1 component was firmly associated with the physical properties of stimuli [75,79]. Anthropomorphic robots with a high degree of anthropomorphism frequently possess facial features that are highly anthropomorphized, leading individuals to experience a sense of uncanniness, which is characterized by perceived negativity. On the other hand, robots with a low degree of anthropomorphism often exhibit non-human characteristics that are more adorable, resulting in individuals perceiving a feeling of cuteness, which is associated with perceived positivity. In contrast, robots with a moderate level of anthropomorphism tend to be perceived as neutral, displaying a moderate level of anthropomorphic traits. Therefore, it is postulated that there would be a higher amplitude of N1 for both high and low anthropomorphic robots, whereas a lower amplitude of N1 is expected for middle anthropomorphic robots.

#### 2.3.2. The P2 Component

The P2, a frontal or occipital-parietal positivity, is involved in higher-level perceptual and attentional processing of visual stimuli [79,80] and has revealed that larger amplitudes can be elicited by emotional stimuli than by neutral stimuli [70,81,82]. Studies have previously shown that P2 is indicative of the early rapid detection of perceptual features and has been reported to be associated with the attentional allocation in the early time window post-stimulus onset [83,84], and it might be modulated by negative or threatening information [85,86]. In addition, the P2 component has been indexed to the allocation of attentional resources for the inherent affective preference in response to stimuli with different perceptual features. For instance, Guo et al. [37] have suggested that an increased magnitude of P2 can be elicited by the preferred appearance of humanoid robots compared to the non-preferred one. Ma et al. [87] have indicated that enhanced P200 can be evoked by the most-preferred product designs of experience goods than for the least-preferred designs. Due to the physical properties and emotional salience, robots with high levels of anthropomorphism frequently possess facial features that are highly anthropomorphized, which may elicit a non-preferred feeling compared to robots with low and middle levels of anthropomorphism. Thus, we expected to see larger P2 for middle and low anthropomorphic robots than for high anthropomorphic robots, reflecting increased selective attention and feature detection.

#### 2.3.3. The LPP Component

The LPP, a positive potential with wide scalp distribution, has been linked to the stimuli’s arousal and valence level, indicating it can reflect individuals’ subjective affective experience [76,88,89,90,91]. Specifically, increased LPP could be evoked by stimuli with high or low valence, and larger LPP could be elicited by higher-arousal stimuli compared to low-arousal stimuli [70,89,91,92,93]. Both positive and negative stimuli could evoke a stronger LPP response than neutral ones [70,76]. Vaitonytė et al. [40] suggested that LPP was susceptible to facial appearance at the temporal level. Convincing evidence also has revealed that the LPP component is associated with the distribution of sustained attention [89], top-down processing [94], affective evaluation [95], categorization [92], and even affective evaluative categorization in preference [73,87]. 

#### 2.3.4. ERSP of Theta Band

In addition to ERPs, time-frequency measurements of neuro-oscillatory power, broadly used in substantial studies concerning the attentional aspects [96,97,98], have been effective in characterizing neural component processes when ERPs may not be interpreted clearly. The oscillations in theta band of emotional stimuli and affective pictures have been related to the distribution of attentional resources in the early perceptual processing [96,97], encoding [99], emotional discrimination [100], evaluation [37], and categorization in the late time window [101,102]. Generally, increased theta power as event-related synchronization (ERS) can be elicited by both positive and negative stimuli rather than neutral stimuli. Convincing evidence has also suggested that theta-band activations are linked with affective preference formation and can reflect the attentional distributions for the affective information processing and evaluative categorization in affective preference formation [37,98]. Due to the physical features and emotional salience, we expected to see greater theta oscillations for high and low anthropomorphic robots than for middle anthropomorphic robots.

We have formulated the following hypotheses based on existing literature (see Table 1):

1. Biomarkers:

**Hypothesis** **1.1.** *Higher anthropomorphism levels will elicit more negative N1 and increased frontal theta power in the early time window*.

**Hypothesis** **1.2.** *Medium and low anthropomorphism levels will induce larger P2 responses compared to high anthropomorphism*.

2. Emotional affect:

**Hypothesis** **2.1.** *High anthropomorphism will result in greater affective arousal as indicated by enhanced LPP*.

**Hypothesis** **2.2.** *Medium anthropomorphism will cause more pronounced parietal-occipital theta-band oscillations than low and high anthropomorphism*.

3. Level of robot anthropomorphism:

**Hypothesis** **3.** *Different levels of anthropomorphism will show distinct neural patterns in both early feature detection and later affective appraisal phases*.

**Table 1:** The summary of hypotheses.

Variables	Low Anthropomorphic Robots (L-AR)	Middle Anthropomorphic Robots (M-AR)	High Anthropomorphic Robots (H-AR)
Biomarkers			
N1			More Negative frontal and central N1
Frontal theta power			Increased
P2	Larger P2	Larger P2	Decreased P2
Emotional affect			
Affective arousal (LPP)		Greater LPP	Greater affective arousal
Parietal-occipital theta		Enhanced	
Overall neural patterns	Distinct patterns in early detection and later appraisal phases	Distinct patterns in early detection and later appraisal phases	Distinct patterns in early detection and later appraisal phases

## 3. Method

### 3.1. Participants

A total of 42 college students (22 males and 20 females; aging from 21 to 25 years; mean age, 22.12 years, SD = 1.24) participated in the study. All were right-handed and reported normal or corrected to normal vision and no history of neurological or psychiatric diseases. The participants were recruited from the undergraduate and graduate populations. Participants were not addicted to drugs or alcohol and were asked to avoid the use of stimulants (e.g., alcohol and caffeine) 48 h before the experiment, which may affect EEG results. All participants provided their written informed consent prior to the experiment and received compensation of 80 RMB or course credits for their participation. Approval for the experiment was obtained from the Ethics Committee of Southeast University. The experiment gathered EEG data from all 42 subjects, two of whom were excluded from the analysis due to excessive artifacts resulting from frequent leg or hand jitters. Thus, the total of subjects included in every grand average for further analysis was 40 (21 males and 19 females; aging from 21 to 25 years; mean age, 22.32 years, SD = 1.38). 

### 3.2. Stimuli

**Figure 2:**
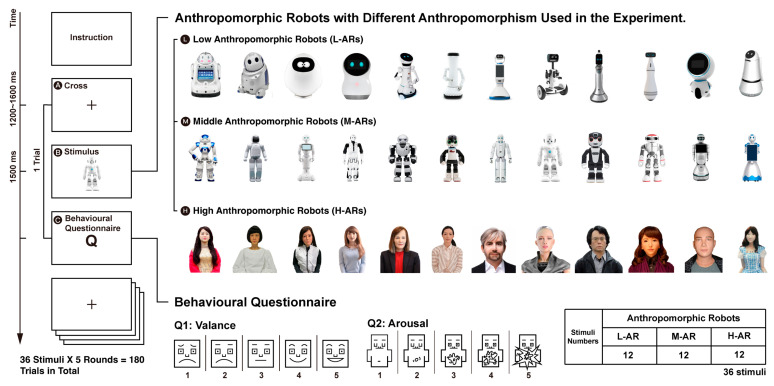
Schematic time course of the experimental procedure and the stimuli used in the experiment.

Most experimental stimuli (Figure 2) were selected from the ABOT (Anthropomorphic roBOT) Database, a collection of 251 images of real-world robots with one or more human-like appearance features [4]. It has been identified that the human-like robot appearance can be divided into four distinct dimensions: surface look, body manipulators, facial features, and mechanical locomotion [4]. An overall human-likeness score for each robot could be computed based on the above four dimensions using the Human-Likeness Estimator proposed by Phillips et al. [4]. According to the human-likeness scores, the initial stimuli were split into three groups, high anthropomorphic robot group (“H-AR”; with human-likeness scores ranging from 80 to 100), middle anthropomorphic robot group (“M-AR”; with human-likeness scores ranging from 40 to 65), and low anthropomorphic robot group (“L-AR”; with human-likeness scores ranging from 0 to 20). Thus, we had sixty-one L-ARs, forty-four M-ARs and seven H-ARs. Due to the lack of H-ARs, we collected an additional 13 H-ARs from the Internet, whose human-likeness scores varied from 88 to 96. Hence, we obtained 20 high-human-likeness robots in total. Then, we asked five Ph.D. candidate volunteers who are proficient in human factors and robot research but did not participate in the late EEG research to classify and rate anthropomorphic robots for each group based on the appearance similarities or shared characteristics among each other on a 5-point Likert scale (1 = not similar at all, 5 = highly similar). We selected 12 stimuli with approximate similarity scores (M_L-AR_ = 4.03; M_M-AR_ = 4.12; M_H-AR_ = 3.38) for each group (see Figure 2), respectively. Finally, we used the one-way ANOVA analysis to compare the human-likeness scores between the three groups. The results showed a significant difference between the groups (M_L-AR_ = 14.340, SD_L-AR_ = 3.968; M_M-AR_ = 47.584, SD_M-AR_ = 5.841; M_H-AR_ = 92.055, SD_H-AR_ = 2.310; *p* < 0.001). 

### 3.3. Procedures

This study was conducted in the Human Factor Engineering Lab at Southeast University with soft lighting, suitable temperature, and noise strictly controlled. The participants were asked to sit in the chair comfortably at 650 mm away from a computer screen. All stimuli (Figure 2) were presented on a 27-inch LCD monitor with a brightness of 92 cd/m^2^, and a resolution of 1920 × 1080 pixels. The experimental task (as shown in Figure 2) was programmed through E-Prime 2.0 (Psychology Software Tools). 

Before the formal experiment, the participants were asked to read the instructions provided. It is worth noting that participants have been briefed on the meanings of emotional valence and arousal during the instruction period. They were also asked to maintain their attentional focus when the fixation cross appeared until the pictures of different anthropomorphic robots disappeared. Once the participants finished reading the instructions provided, the formal experimental procedure began. Each trial began with a fixation cross that remained in the center of the screen for 1200–1600 ms to prime individuals for the EEG experiment. This was followed by the presentation of different anthropomorphic robot stimuli for 1500 ms. After viewing and perceiving the experimental stimuli, the participants were presented with the following behavioral questionnaire: “What do you think is the level of emotional valence produced by the anthropomorphic robot?” and “What do you think is the level of emotional arousal produced by the anthropomorphic robot?”. The options for responses were designed by fusing the Likert 5-point scale with the Self-Assessment Manikin (SAM) [103], where ‘1’ represents the lowest level and ‘5’ represents the highest level. Participants were required to finish the two questionnaires by using a keyboard. Subsequently, the next trial started. A total of 180 trials were presented in five blocks, with the order of trials within each block randomized. The experimental stimuli appeared randomly to eliminate the sequential effect. A rest period was given after each block, and the length of the rest interval was self-determined by the participants before continuing the experiment. Each participant spent approximately 40 min completing the experiment. After the experiment, participants were asked to finish a likeability questionnaire [63] (Cronbach’s alpha is well above 0.84). To facilitate comprehension, the likeability scale was modified into a more accessible Likert 5-point scale. Each word (讨人喜欢的/likable, 令人感到亲切的/friendly, 待人礼貌的/kind, 令人愉快的/pleasant, 让人放心接近的/approachable) was divided into five levels [42,63]. Subsequently, the human warmth questionnaire [13] (Cronbach’s α = 0.95) was presented with five warmth-related traits (合群的/sociable, 令人感到亲切的/friendly, 充满善意的/kind, 可爱的/likable, 充满温情的/warm) in the manner of the Likert 5-point scale, and the participants were asked to response how close the descriptions were to their own feelings.

### 3.4. Electroencephalogram Data Recordings and Preprocessing

**Figure 3:**
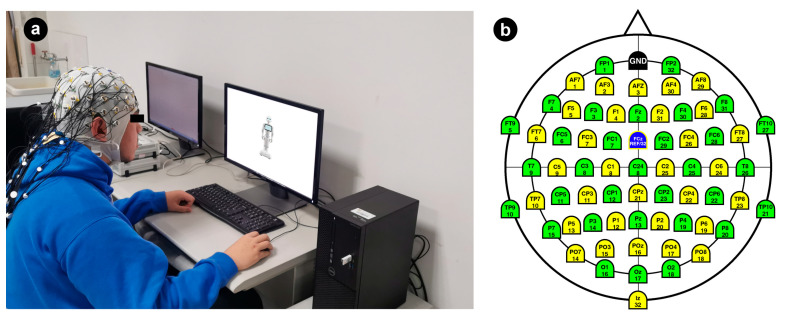
Experimental equipment set up and electrodes. (**a**) Brain vision actiCHamp EEG system; (**b**) 64 electrodes (with names and number labels) used in the experiment.

The EEG data were continuously recorded (bandpass 0.05–100 Hz, at a 1000 Hz sampling rate) by the Brain Vision actiCHamp EEG system (Brain Product, Munich, Germany) (Figure 3a) with 64 Ag/AgCl electrodes (Figure 3b). The electrodes were mounted on an elastic electrode cap based on the international 10–20 system [104], and the sixty-four channels were all utilized for recording the EEG signals. The FCZ electrode was utilized as the reference electrode, and FPZ served as the ground electrode. Interelectrode impedances of all electrodes were kept below 5 kΩ throughout the experiment. 

**Figure 4:**
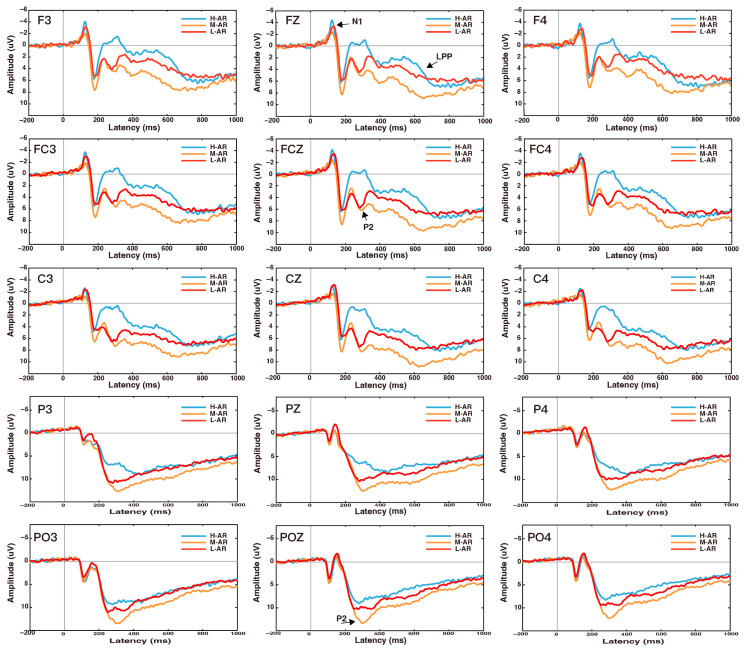
The grand-averaged ERP waveforms in response to high, middle, and low anthropomorphic robots.

The EEG data were preprocessed offline in MATLAB 2013b (MathWorks Inc., Natick, MA, USA) using the EEGLAB 13 toolbox (Swartz Center for Computational Neuroscience, UCSD; http://sccn.ucsd.edu/eeglab (accessed on 5 June 2023). The raw EEG recordings were re-referenced to the average of the TP9 and TP10 channels, down-sampled to 500 Hz and were filtered through a 30-Hz low-pass and 0.1-Hz high-pass filter, respectively. Segments with a low signal noise ratio (SNR) were then excluded and independent component analysis (ICA) was performed. Artifacts (e.g., electro-oculogram (EOG), electromyogram (EMG), and sweat) were corrected by employing the ADJUST1.1.1 plugin in the EEGLAB toolbox. The EEG data were epoched from 200 ms prior to the onset of the anthropomorphic robots to 1000 ms after the presentation, with the first 200 ms interval serving as the referent baseline. Epochs of each trial type were then categorized. Epochs with amplitudes exceeding ±80 μV threshold were rejected [105,106]. z-score normalization was performed on the ERP amplitudes across subjects, converting the amplitudes into standard scores. These steps ensure that the ERP data are comparable across subjects, mitigating potential biases due to scale differences [79]. After rejection, at least 56 trials per participant were available for each type of anthropomorphic robot. The rejection rates for H-AR\M-AR\L-AR were 6.67%, 5% and 3.33%, respectively. There were no significant group differences in the rejection rates (F (2, 57) = 0.355, *p* = 0.703). The grand average waveforms for different anthropomorphic robots are depicted in Figure 4. 

### 3.5. Data Analysis

#### 3.5.1. ERP Analysis

**Figure 5:**
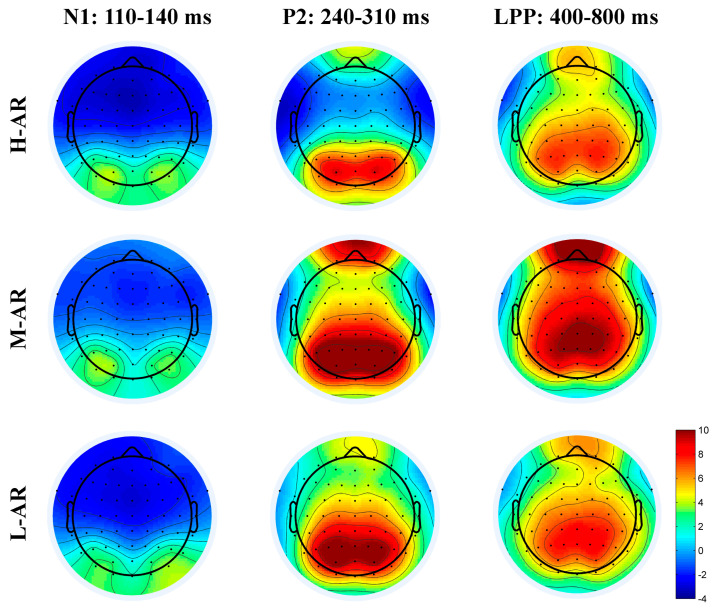
Topographic scalp maps of ERPs component during selected time course for high, middle, and low anthropomorphic robots.

As shown in Figure 4 and Figure 5, the N1 (110–140 ms), P2 (240–310 ms) and LPP (400–800) have been elicited in different anthropomorphic levels of robots. Based on the visual inspection, the N1 was more obvious in the anterior sites, while P2 and LPP components were more pronounced in the anterior and posterior sites. Thus, three electrode clusters, including the frontal (F3, FZ, F4), frontal-central (FC3, FCZ, FC4), and central (C3, CZ, C4) regions were selected for N1 component analysis. The P2 and LPP component analysis was performed at six electrode clusters including the frontal (F3, FZ, F4), frontal-central (FC3, FCZ, FC4), central (C3, CZ, C4), central-parietal (CP3, CPZ, CP4), parietal (P3, PZ, P4) and parietal-occipital (PO3, POZ, PO4) locations. The averaged amplitude of N1 in the time window of 110–140 ms was analyzed in a 3 (Type of robot: high, middle, and low anthropomorphic robots; H-AR\M-AR\L-AR) × 3 (Location: frontal, frontal-central, central; F, FC, C) two-way ANOVA. The averaged amplitude of each ERP component (P2 and LPP) was analyzed in a 3 (Type of robot: high, middle, and low anthropomorphic robots; H-AR\M-AR\L-AR) × 6 (Location: frontal, frontal-central, central, central-parietal, parietal and parietal-occipital; F, FC, C, CP, P, PO) two-way ANOVA. If necessary, the Greenhouse–Geisser corrections of freedom were applied when the data failed the sphericity tests, and the Bonferroni correction was employed for post hoc testing as needed. 

#### 3.5.2. Event-Related Spectral Perturbations (ERSPs) Analysis

We reprocessed the EEG raw data with a 0.15 Hz high-pass filter. Other preprocessing processes were similar to ERP data analysis, except a longer epoch for each trial type from 500 ms pre-stimulus onset to 1500 ms post-stimulus onset was segmented for further time-frequency analysis. To gain a comprehensive view of neural oscillations across the scalp of anterior and posterior sites, time-frequency decomposition was carried out on the two electrode channel clusters, frontal cluster (AF3, AFZ, AF4, F3, FZ, F4, FC3, FCZ, FC4) and parietal-occipital cluster (CP3, CPZ, CP4, P3, PZ, P4, PO3, POZ, PO4), each of which was an average of selected channels. These channels were chosen mainly based on visual inspection of topographic maps and prior work. Each epoch was split into 200 time points ranging from −372 ms to 1372 ms. The signals of EEG data were decomposed by short-time Fourier transformation with Hanning window tapering as implemented in the EEGLAB function *newtimef.m*. Using a sliding window of 256 ms with a step size of 10 ms and a filling ratio of 4 (default value), the spectral power at each time point in each EEG epoch was calculated, yielding 48 linear-spaced frequencies ranging from 3 to 50 Hz [107]. Subsequently, baseline correction was implemented according to the gaining model [108], and the spectral power of each time-frequency point was divided by the mean pre-stimulus baseline power of the corresponding frequency. Finally, the spectral power at each time point and frequency were then averaged for all segments within each trial type of each cluster. ERSPs were analyzed in the early time window (50–380 ms) and the late time window (400–1000 ms) for the two-channel clusters within the theta band (3–8 Hz). Theta band was selected based on previous work which suggests that theta band is associated with attentional distribution and affective preference [37,96,98]. The theta power was analyzed using a 3 (Type of robot: high, middle, and low anthropomorphic robots; H-AR\M-AR\L-AR) × 2 (Cluster: frontal, parietal-occipital) analysis of variance (ANOVA). The Greenhouse–Geisser and Bonferroni corrections were employed whenever necessary. 

#### 3.5.3. Statistical Analysis

All the statistical analyses of subjective ratings, ERP, and ERSPs data were performed in IBM SPSS Statistics 26.0. The normality of the data was verified by using the Kolmogorov–Smirnov test, and the results indicated that the data were normally distributed, and the variance was homogenous (*p* > 0.05). If necessary, the Greenhouse–Geisser corrections of freedom were applied, and the Bonferroni correction was employed for post hoc testing. The alpha level was set as 0.05 for statistical tests.

## 4. Results

### 4.1. Results for Subjective Rating Data

**Figure 6:**
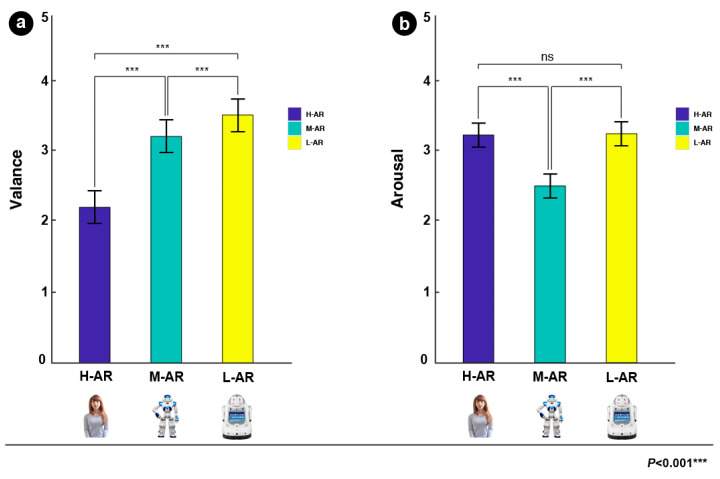
Figure (**a**) and figure (**b**) showed emotional valence and emotional arousal results of the 40 participants toward the three types for anthropomorphic robots, respectively.

**Emotional valence and arousal ratings:** The analysis revealed that the type of anthropomorphic robots had significant effects on the rating of the emotional valence (F (2, 40) = 87.294, *p* < 0.001, $\eta_{p}^{2}$ = 0.599), and the emotional arousal (F (2, 40) = 5.849, *p* = 0.005, $\eta_{p}^{2}$ = 0.170). Further multiple comparisons (Figure 6a) revealed that L-AR had higher mean valence scores (M = 3.499, SD = 0.393) than H-AR (M = 2.194, SD = 0.599) (*p* < 0.001) and M-AR (M = 3.089, SD = 0.314) (*p* < 0.001), and the averaged valence of M-AR was greater than H-AR (*p* < 0.001). The averaged arousal scores of both L-AR (M = 3.230, SD = 0.707) and H-AR (M = 3.223, SD = 0.707) were significantly larger than M-AR (M = 2.510, SD = 0.409) (Figure 6b). 

**Figure 7:**
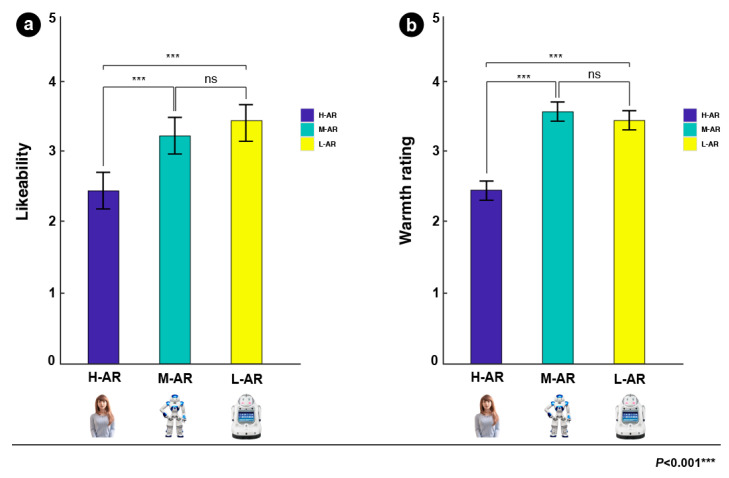
Figure (**a**) and figure (**b**) showed the subjective likeability and warmth rating results of the 40 participants toward the three types for anthropomorphic robots, respectively.

**Likeability and warmth ratings**: Results on the likeability of different anthropomorphic robots showed a significant effect (F (2, 40) = 31.829, *p* < 0.001, $\eta_{p}^{2}$ = 0.352). The averaged likeability of L-AR (M = 3.421, SD = 0.385) was larger than H-AR (M = 2.429, SD = 0.866) (*p* < 0.001) and M-AR (M = 3.263, SD = 0.406) (*p* = 0.238), and averaged likeability of M-AR was significantly larger than H-AR (*p* < 0.001) (see Figure 7a). There was also a significant effect on warmth scores of the type of robots (F (2, 40) = 16.484, *p* < 0.001, $\eta_{p}^{2}$ = 0.220). The mean warmth rating of H-AR (M = 2.404, SD = 0.563) was smaller than M-AR (M = 3.641, SD = 0.349) (*p* < 0.001) and L-AR (M = 3.534, SD = 0.394) (*p* < 0.001), whereas no significant difference (Figure 7b) between M-AR and L-AR was observed (*p* = 0.840).

### 4.2. ERP Results

#### 4.2.1. N1 Component (110–140 ms)

ANOVA results of N1 showed significant main effects of type of robot (F (2, 78) = 7.514, *p* = 0.001, $\eta_{p}^{2}$ = 0.162), and location (F (1.159, 45.191) = 16.927, *p* < 0.001, $\eta_{p}^{2}$ = 0.303). Besides, there was a significant interaction between the type of robot and location (F (2.379, 92.774) = 4.699, *p* = 0.008, $\eta_{p}^{2}$ = 0.108). Pairwise comparisons showed significant differences between types of robots, with larger negative amplitude for H-AR (M = −2.726, SE = 0.367) and L-AR (M = −2.513, SE = 0.362) than for M-AR (M = −1.410, SE = 0.437) (H-AR > M-AR, *p* = 0.005; L-AR > M-AR, *p* = 0.025). Bonferroni post hoc multiple comparisons (see Table 2) demonstrated that H-AR and L-AR elicited negative amplitude than M-AR at frontal and frontal-central locations than at central sites (F > C, *p* = 0.001; FC > C, *p* < 0.001). Regarding latency of the N1 component, there was also a significant main effect of the type of robot (F (2, 78) = 4.858, *p* = 0.01, $\eta_{p}^{2}$ = 0.111). Compared to M-AR, the N1 peaked earlier for H-AR.

#### 4.2.2. P2 Component (240–310 ms)

In the time course of 240 ms–310 ms after the stimuli, the main effects of types of robots (F (2, 78) = 53.392, *p* < 0.001, $\eta_{p}^{2}$ = 0.600) and location (F (1.211, 47.217) = 55.613, *p* < 0.001, $\eta_{p}^{2}$ = 0.588) arrived at significance. Also, there was a significant interaction between the type of robot and location (F (2.571, 100.282) = 20.057, *p* < 0.001, $\eta_{p}^{2}$ = 0.340). Pairwise comparisons revealed significant differences between types of robots, with greater amplitude for M-AR (M = 7.946, SE = 0.695) than for L-AR (M = 6.927, SE = 0.672) and H-AR (M = 3.182, SE = 0.537) (M-AR > L-AR, *p* < 0.001; M-AR > L-AR, *p* = 0.021; L-AR > H-AR, *p* < 0.001). Type simple effects were significant for frontal (F (2, 78) = 41.62, *p* < 0.001), frontal-central (F (2, 78) = 61.78, *p* < 0.001), central (F (2, 78) = 68.39, *p* < 0.001), central-parietal (F (2, 78) = 69.57, *p* < 0.001), parietal (F (2, 38) = 45.92, *p* < 0.001) and parietal-occipital (F (2, 78) = 24.96, *p* < 0.001) sites. 

#### 4.2.3. LPP Component (400–800 ms)

Statistical analysis of the LPP component revealed the significant main effects of the type of robot (F (2, 78) = 15.816, *p* < 0.001, $\eta_{p}^{2}$ = 0.289) and location (F (1.254, 48.916) = 12.926, *p* < 0.001, $\eta_{p}^{2}$ = 0.249), and their interaction effect achieved significance (F (2.858, 111.473) = 4.828, *p* = 0.004, $\eta_{p}^{2}$ = 0.110). Pairwise comparisons revealed significant differences between types of robots, with greater amplitude for M-AR (M = 8.402, SE = 0.954) than for L-AR (M = 6.371, SE = 0.967) and H-AR (M = 5.764, SE = 0.749) (M-AR > L-AR, *p* < 0.001; M-AR > L-AR, *p* < 0.001). Type simple effects were significant for frontal (F (2, 78) = 18.65, *p* < 0.001), frontal-central (F (2, 78) = 17.92, *p* < 0.001), central (F (2, 78) = 16.91, *p* < 0.001), central-parietal (F (2, 78) = 14.80, *p* < 0.001), parietal (F (2, 78) = 10.32, *p* < 0.001) and parietal-occipital region (F (2, 78) = 7.93, *p* = 0.001). 

### 4.3. ERSP Results

ANOVA results of theta power across the early time window (50–380 ms) revealed that only the main effect of the cluster was statistically significant (F (1, 39) = 20.931, *p* < 0.001, $\eta_{p}^{2}$ = 0.349), with power being greater for the parietal-occipital cluster (M = 2.571 dB, SE = 0.111) than for the frontal cluster (M = 2.044 dB, SE = 0.091). No significant main effect of the type of robot was observed (F (2, 78) = 1.213, *p* = 0.303, $\eta_{p}^{2}$ = 0.030). There was a significant interaction between the type of robot and cluster (F (2, 78) = 4.182, *p* = 0.019, $\eta_{p}^{2}$ = 0.097). Post hoc analyses revealed that theta power of H-AR (M = 2.214 dB, SE = 0.136) and M-AR (M = 2.229 dB, SE = 0.186) was larger than that of L-AR (M = 1.689 dB, SE = 0.151) (*p* = 0.044) in the frontal cluster. 

**Figure 8:**
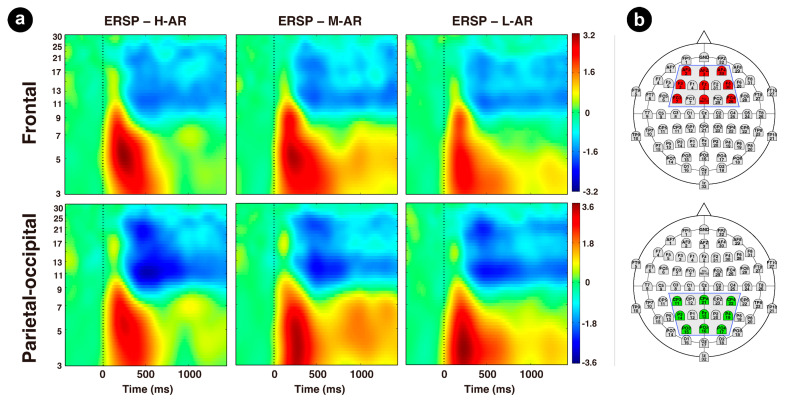
Spectrograms of theta-band (3–8 Hz) ERS at the frontal cluster and parietal-occipital cluster associated with H-AR\M-AR\L-AR conditions: (**a**) the time–frequency representations of ERD/ERS related to H-AR\M-AR\L-AR conditions at the frontal and parietal-occipital clusters and (**b**) the channel electrode clusters of interest. The red represents frontal cluster, while the green represents parietal-occipital cluster.

**Figure 9:**
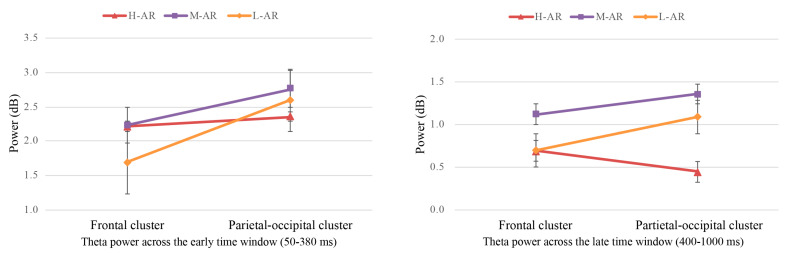
The (**left**) and (**right**) plots represented the interaction effect of theta power of frontal cluster and parietal-occipital cluster across the early time window (50–380 ms) and the late time window (400–1000 ms), respectively.

Analysis of the late time window (400–1000 ms) showed that the main effect of the type of robot was significant (F (2, 78) = 5.256, *p* = 0.007, $\eta_{p}^{2}$ = 0.119), with ERSP being greater for M-AR (M = 1.236 dB, SE = 0.181) than for H-AR (M = 0.573 dB, SE = 0.114) and L-AR (M = 0.893 dB, SE = 0.170). No main effect of cluster achieved significance (F (1, 39) = 2.438, *p* = 0.126, $\eta_{p}^{2}$ = 0.059). There was a significant interaction between the type of robot and cluster (F (2, 78) = 8.633, *p* < 0.001, $\eta_{p}^{2}$ = 0.181). Simple effect analysis revealed that the perturbations of H-AR (M = 0.453 dB, SE = 0.154) were smaller than M-AR (M = 1.353 dB, SE = 0.139; *p* < 0.001) and L-AR (M = 1.087, SE = 0.188; *p* = 0.022) in the parietal-occipital cluster (see Table 3 for group means, Figure 8 for spectrograms and Figure 9 for interaction effect of theta power).

### 4.4. Correlations between Emotional Responses, ERPs, and ERSP

**Figure 10:**
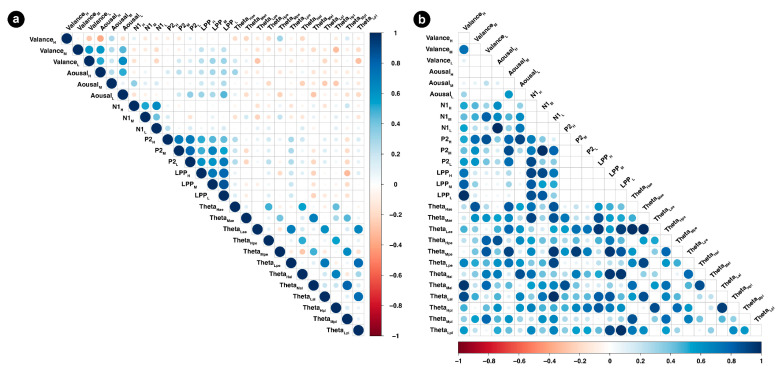
(**a**) represents Spearman’s r of emotional responses, ERPs and ERSP. (**b**) represents Spearman’s p of emotional responses, ERPs and ERSP. The statistical method used the Spearman correlation coefficient; a and p indicate the frontal and posterior regions, respectively, e and l represent the early time window and late time window, respectively.

The results of correlation analyses after Bonferroni correction are shown in Figure 10. The findings revealed that L-AR valence had significant positive correlations with L-AR arousal. The valence of H-AR correlated negatively with H-AR arousal. P2 and LPP of L-AR both had positive correlations with L-AR arousal. The P2 of H-AR had significant positive correlations with the LPP of H-AR, and the P2 of M-AR correlated significantly positively with the LPP of M-AR. Notably, we discovered that the frontal theta power of H-AR, M-AR, and L-AR all had significant positive correlations with both early and later theta rhythm power in parieto-occipital regions.

## 5. Discussion

Robots are becoming more prevalent in human social life and play a significant role in a range of industries. The emotional experience prompted by the visual appearance of an anthropomorphized robot plays a critical role in affecting users’ behaviors. Moreover, individual’s attitudes towards robots with varying degrees of anthropomorphism are also important. The purpose of this study is to tackle the time course of neural processing of human perceptions and emotional responses on three different types of robots (H-AR\M-AR\L-AR) with varying levels of anthropomorphism, and to evaluate individual’s subjective emotional valence and arousal, as well as attitudes, such as likeability, and perceived warmth toward the stimuli. This research combined subjective ratings, ERPs and ERSP measurements to characterize neural cognitive process components. The findings might contribute to a better understanding of users’ perceptual and emotional processing of anthropomorphized robots and facilitate the design of emotional anthropomorphic robots.

### 5.1. Behavioral Results Discussion

The subjective rating results manifested that the emotional valence is negatively correlated with the level of the robot’s anthropomorphism, while emotional arousal is accompanied by a U-shaped function of anthropomorphism, with higher arousal at the low anthropomorphic level, followed by a decrease at middle level and finally by a re-increase in arousal at high level. Consistent with Bradley and Lang [109], our results suggested that low anthropomorphic levels may induce positive emotional experiences and extreme emotional valence is often related to high emotional arousal for positive or negative stimuli. Regarding the likeability, the results showed that the likeability of M-AR was larger than H-AR, which is in line with previous studies [14,15,19]. These studies suggested that likeability increased with the increase in human likeness, whereas they would be perceived as really unlikeable and induce negative attitudes as robots became more human-like [10,14]. However, in the present study, we also found that L-AR has higher likeability than M-AR. The likely account for this might be that the experimental sample of L-AR includes more adorable non-human characteristics such as eyes, legs, or arms, compared to M-AR stimuli. In addition, the L-AR can be perceived as less threatening to human distinctiveness relative to M-AR. For human warmth, the averaged warmth rating was smallest during the H-AR condition, while the warmth means were relatively close between M-AR and L-AR conditions. The results were partially consistent with the findings of Kim et al. [13], suggesting that once robots become too human-like, an uncomfortable feeling of uncanniness could appear and lead to less positive attitudes. The slight difference between L-AR and M-AR might be because the experimental stimuli for L-AR were totally different from the experimental stimuli (Ethon 2) in Kim’s study, the stimuli for L-AR and M-AR in the current study all had likable product shapes and distinctive characteristics of appearance. Thus, L-AR might have a perceived warmth that is comparable to M-AR. These findings suggest that the degree of anthropomorphism of robots may play an important role in affecting users’ perceptual and emotional processing, as well as judgments of robots.

### 5.2. ERPs Related to Anthropomorphic Robots

#### 5.2.1. N1 (110–140 ms): An Early Perceptual Detection of Anthropomorphic Robot Features (Hypotheses 1.1 and 3)

Consistent with prior emotional ERPs research that affective stimuli can elicit an enhanced N1 component compared to neutral stimuli [70,76,77], in the present study N1 amplitudes of H-AR (high arousal and low valence) and L-AR (high arousal and high valence) were significantly larger negative than that of M-AR (middle arousal and middle valence) in the anterior sites (supported H1.1 and H3). The possible explanation could be that H-AR and L-AR with high arousal scores may be more inclined to draw more attention from participants in the early information processing stream. Furthermore, a large number of studies on robot design have confirmed that human likeness [110], appearance [37], actions [22,24], and automation [26] can have an influence on a user’s perceptual process, attentional distribution, preference, and even the following behaviors. Prior ERP studies have found that the N1 component is firmly related to the physical properties of events [75,79], and it has also been associated with selective attention and discrimination [71,73,111]. In the present study, H-AR and L-AR all exhibit relatively prominent appearance compared to M-AR. Thus, both H-AR and L-AR received enhanced attention allocation. As 125 ms is a very early time point and the information processing at this phase probably occurred subconsciously, thus the nervous system might only detect a few features of stimulus pictures. We also observed shorter N1 latencies for H-AR than for L-AR. H-AR was perceived as more human-like compared with L-AR. H-AR with highly anthropomorphic faces might be more cognitively accessible and anthropomorphized during the face processing stage. Thus, H-AR might attract individuals’ attention more quickly for face form detection relative to L-AR in the early phase of perceptual processing (partially supported 3).

#### 5.2.2. P2 (240–310 ms): A Selective Attentional Allocation of Anthropomorphic Robot Features (Hypotheses 1.2 and 3)

Previous studies have shown that P2 enhancement will be elicited by stimuli with positive or negative valence compared to stimuli with neutral valence [82]. In the present study; however, larger P2 amplitudes were observed for the M-AR and L-AR than for the H-AR over a wide region across the scalp, and M-AR has greater P2 amplitudes compared with L-AR in the parietal and occipital regions (partially supported H1.2 and H3). Prior research has revealed that P2 is sensitive to early stimulus classification and reflects the early attentional bias to the characteristics of the stimulus itself [85,112]. Several previously reported studies have also shown that natural selective attention can occur and account for the variation in amplitudes during middle latency [62,78,91]. These studies suggested that some features of the stimulus itself may be more emotionally stimulating in users, prompting them to allocate more or earlier attention to the stimulus pictures. We tentatively interpreted the above-mentioned results could be attributed to the different physical properties. In the present study, H-ARs (high arousal) were much more human-like and had a high level of anthropomorphism with more prominent characteristics of humans (with half-bodies), thus H-AR might be more cognitively accessible, more sought by perceivers, and could be identified or distinguished rapidly and automatically by users relative to L-AR and M-AR. Both M-AR and L-AR with relatively moderate or less prominent characteristics of human beings need users to give more effort and attention to acquiring information for the affective evaluation. Accordingly, P2 amplitude was more strongly activated by M-AR and L-AR than that by H-AR. Consequently, smaller P2 amplitudes of H-AR are probably indicative of a feature detection process that is responsive to high levels of anthropomorphism. In addition, relative to L-AR and M-AR, H-AR with more prominent humanlike design characteristics was perceived as much more human-like and also can give individuals a potentially weird, uncomfortable feeling of uncanniness (participants’ post hoc behavioral response supported this) [10,13,43,56,113], which might recruit participants’ more or earlier attentional resources to respond to stimuli rapidly and automatically [114,115]. Thus, in the current study, the face form detection and animacy perception of H-AR might be facilitated and finished in this time course [56,116,117]. Moreover, the P2 peaked earlier for H-AR than for L-AR and M-AR, suggesting that individuals can have a faster feature detection for high anthropomorphic stimuli relative to low ones. In congruence with Chammat et al. [20], this study suggests that the appearance of humanoid robots (L-AR and M-AR) can engage more attentional resources in detecting and encoding. Noticeably, the P2 of M-AR is greater in posterior areas than that of L-AR, while no difference has been found between M-AR and L-AR in the anterior regions. The main reason for this was that M-AR may recruit enhanced attentional resources for the working memory of encoding in posterior areas compared to the L-AR condition, whereas the L-AR and M-AR devoted approximative attentional resources for the rapid feature detection in frontal regions.

#### 5.2.3. LPP (400–800 ms): An Affective Evaluation, Categorization and Motivated Attention of Anthropomorphic Robots (Hypotheses 2.1 and 3)

Prior research has demonstrated that LPP is linked to the stimuli’s arousal and valence level [76,88,90,93,118]. In contrast to neutral stimuli, stimuli with high or low valence could elicit enhanced LPP, and stimuli with higher arousal scores could evoke greater LPP than low-arousal stimuli [70,89,92]. However, in the present study, larger LPP (during the 400–800 ms interval) was elicited by M-AR than by L-AR and H-AR, while M-AR held low arousal and middle valence ratings (participants’ affective evaluation results) relative to L-AR and H-AR (not supported H2.1, but supported H3). The difference might be attributed to the experimental stimuli used in the study, which held different levels of anthropomorphism and appeared to induce distinguished neural patterns. Moderately human-like appearances appeared to be not fearful or ugly enough to evoke negative emotional experiences [37,119]. LPP has previously been reported to be associated with sustained attention allocation, top-down processing influences, evaluation of emotional stimuli, subjective affective experience, and categorization processes [40,78,79,89,118,120,121,122,123]. In the current study, when participants make an affective evaluation of the picture stimuli and then give the corresponding emotional valence and arousal scores, more attentional resources and more heavily weighted evaluative judgments might be motivationally assigned to M-AR caused by distinct physical attributes (i.e., relatively and moderately anthropomorphic characteristics, such as eyes, legs, and face) through the top-down modulation, inducing a higher level of arousal. LPP enhancement for M-AR might reflect the top-down control and motivated attention needed for the affective evaluation. Furthermore, this result was also in agreement with the findings of Jacobsen and Höfel [124], suggesting that aesthetic discrimination of preference induced sustained LPP. Evaluating M-AR appears to activate the arousal of inherent affect in preference formation for human-like appearances [37], and the association of knowledge in long-term memory and involves top-down processing. This may have contributed more to positive emotion. Thus, significantly greater LPP was observed for M-AR than for L-AR and H-AR. Noticeably, we also found that the LPP for M-AR and L-AR in this study had a wider scalp distribution than for H-AR. The scalp distribution was partially in line with the findings of Wang and Quadflieg [125], who suggested that more cortical regions would be active for the cognitive processing of humanoid robots than for human beings (H-AR was perceived as more human-like). In the same vein, it was also partially consistent with the findings of Cheetham et al. [36], who suggested a lower scalp topographical distribution of the LPP for human and humanlike faces than ambiguous ones.

### 5.3. ERSP Related to Anthropomorphic Robots (Hypotheses 1.1, 2.2 and 3)

Prior studies have shown that early theta-band oscillations are involved in the processing of affective stimuli, formation of affective preference during the early perceptual phase, and encoding of stimuli characteristics in working memory [37,73,96,98,126]. In the present study, greater theta band activation was elicited in the parietal-occipital cluster than for the frontal cluster within the early time window (50–380 ms). A possible explanation for this might be that enhanced allocation of attentional resources has been used for encoding anthropomorphic stimuli in working memory. It appeared that dissociations have occurred between the anterior theta-band oscillations and posterior theta-band oscillations in the mechanisms of visual feature detection and attentional processing they reflect. As defined by the two-stage concept of affect and attention, the information processing stream consists of two stages, in which further affective evaluation and categorization take place in the late stage based on the information processed in the early stage [91,127]. Moreover, studies have reported that the information processing in the late stage was typically modulated by a particular goal and the theta-band oscillations might vary as motivated attention [102,128]. In the present study, participants were required to make an affective evaluation of the picture stimuli and subsequently give the corresponding emotional valence and arousal ratings. With the task target, individuals might further motivationally process the pre-processed information of the early perceptual processing phase according to their own preferences. In the current study, individuals tended to prefer M-AR and L-AR compared with H-AR (not supported H1.1). M-AR and L-AR had larger theta-band ERS than H-AR across the late time window (400–1000 ms) (partially supported H2.2, and supported H3). The result was in accordance with prior studies that preferred appearances could draw more attentional resources with the inherent positive affect and elicit increased theta-band activity [37,129].

### 5.4. Correlations of EEG and Behavioral Measures, and the Two Stages

The results revealed that the valence of L-AR has a positive correlation with arousal, but is negative for H-AR. The possible explanation for this might be attributed to the differences in physical properties. Compared with the L-AR, H-AR appeared to be perceived as much more human-like and tended to give individuals a potentially uncomfortable feeling of uncanniness. The P2 of H-AR and M-AR have significant associations with LPP, suggesting associations between the induced information and perceptual processing reflected in ERP. With regard to the theta power of the two stages, the frontal-cluster theta power of H-AR, M-AR, and L-AR all had significant positive correlations with both early and later theta power in parieto-occipital-cluster regions. The correlations between the frontal-cluster regions and parieto-occipital-cluster regions might indicate the internal correlations of the brain. The correlations between the early time window and the late time window might signify the perceptual information transfers between the early perceptual processing of physical properties and the later further processing and evaluation of preference. This was supported by the two-stage concept of affect and attention [91,127].

### 5.5. Limitations and Future Research

This study has several limitations that should be acknowledged. Firstly, this research used static images, but real-world situations involved dynamic entities with artificially anthropomorphic speech and robot interaction. In the future, we can try to design experiments using dynamic robots or robots with verbal interaction. Secondly, in the current study, only three types of anthropomorphic robots were included, ignoring industrial robots and uncanny valley robots like Zombie and Animated characters. In future research, those robots as factors in the experimental design will be considered. Thirdly, some L-AR had appearances without control of face direction or picture views. The face direction might influence users’ attention direction. And the whole appearances in the L-AR and M-AR look significantly different from the half-bodies of H-AR. This difference may bias the results of this study. Thus, future studies will strictly control these factors. Another limitation of our study is the homogeneous sample consisting primarily of university students familiar with technology. This familiarity may have influenced their perception of the robots, potentially leading to more favorable usability and acceptance outcomes. Future research should consider including a more diverse sample with varying levels of technological exposure to examine how familiarity impacts interaction with robotic devices. Additionally, it would be beneficial to investigate the effects of training and exposure on populations less familiar with technology, as suggested by prior research [130,131]. Fifthly, although the current study provided a potential link between the P2 component and the anthropomorphism, further work at an electrophysiological level was needed to better comprehend the cognitive basis of anthropomorphism, and eventually define and manipulate anthropomorphisms to further explore human cognition and the uncanny valley hypothesis. Finally, ERPs and ERSP were utilized to analyze H-AR\M-AR\L-AR perceptual and emotional processing in time and the time-frequency dimensions. In future research, spatial location analysis for brain functional regions would be measured to complement the experimental results by combining the EEG and fNIRS or fMRI technology.

## 6. Conclusions

Robots are increasingly being used in human social life and play a vital role in numerous fields. The current study used electrophysiological techniques combining ERPs and ERSP to investigate the time course of how the degree of anthropomorphism of anthropomorphic robots affects users’ perceptual and emotional processing, as well as to assess individuals’ attitudes to them. Anthropomorphic robots with three levels of anthropomorphism were used as stimuli in an affective rating task. Forty-two participants viewed, perceived, and rated their emotional scores while EEG data were recorded. 

The behavioral results suggest that emotional valence is negatively correlated with the level of the robot’s anthropomorphism. Emotional arousal is accompanied by a U-shaped function of anthropomorphism. The likeability of L-AR was highest, while the H-AR was lowest. The perceived warmth rating of high anthropomorphic robots is lowest compared to low and middle ones. These findings suggest that the degree of anthropomorphism of robots may play an important role in affecting users’ perceptual and emotional processing, as well as judgments of robots.

The EEG results of the present study suggest that in the early time window, H-AR and L-AR elicited increased exogenous frontal and central N1 than M-AR, reflecting increased attention in the early perceptual processing stage. However, M-AR and L-AR induced enhanced P2 than H-AR, indicating that more selective attention was attracted by M-AR and L-AR. The cognitive underpinnings of the uncanny valley may be indicated by the smaller P2 with peaked earlier latencies for H-AR. At a later stage, M-AR evoked larger LPP than H-AR and L-AR across a wide scape, indicating increased arousal, enhanced directed attention and affective preference. Theta-band ERS results indicate different neural patterns for H-AR, M-AR and L-AR in the early and late time windows. Specifically, theta-band oscillations are greater for the parietal-occipital cluster than the frontal cluster in the early time window, indicating that enhanced attention was used for stimuli information encoding in working memory and a dissociation between the anterior and posterior theta-band oscillations. In the late time window, M-AR and L-AR had larger theta-band ERS than H-AR, indicating enhanced attentional and affective preferred responses. These findings suggest that the degree of anthropomorphism of robots elicited differential neural perceptual and emotional processing of H-AR, M-AR and L-AR, which not only occurred in the early feature detection and selective attentional allocation phase but also took place in later affective appraisal processes. Early face form detection and animacy perception may have been completed in the early time phase, and the P2 related to high-order visual processing may be an indicator associated with the levels of anthropomorphism. These findings imply that robot designers can monitor and understand a user’s perceptual and emotional processing of an anthropomorphic robot based on the neurophysiology components. This may help them better able to design and evaluate anthropomorphic robots that meet consumers’ affective expectations. This study extends anthropomorphic robot design research in emotional processing by using electrophysiological methods. Both robot designers and manufacturers may benefit from this approach, which provides reference and guidelines for the emotional design, feature design, assessment, and promotion of anthropomorphic robots.

## Figures and Tables

**Table 2:** The descriptive statistics and results of ERPs of user’s perception of different anthropomorphic robots.

Locations	Pairwise	*p* of Comparing N1 Component Amplitude	*p* of Comparing P2 Component Amplitude	*p* of Comparing LPP Component Amplitude
Frontal	H–M	0.003 **	<0.001 ***	<0.001 ***
	H–L	0.430	<0.001 ***	1.000
	M–L	0.064	0.205	<0.001 ***
Frontal-central	H–M	0.004 **	<0.001 ***	<0.001 ***
	H–L	1.000	<0.001 ***	0.325
	M–L	0.030 *	0.727	<0.001 ***
Central	H–M	0.017 *	<0.001 ***	<0.001 ***
	H–L	1.000	<0.001 ***	0.271
	M–L	0.010 *	0.800	<0.001 **
Centro-parietal	H–M	-	<0.001 ***	<0.001 ***
	H–L	-	<0.001 ***	0.483
	M–L	-	0.369	0.002 **
Parietal	H–M	-	<0.001 ***	0.001 **
	H–L	-	<0.001 ***	1.000
	M–L	-	0.010 *	0.003 **
Parietal-occipital	H–M	-	<0.001 ***	0.003 **
	H–L	-	0.027 *	0.688
	M–L	-	<0.001 ***	0.013 *

Notes: L, M, and H represented low, middle, and high anthropomorphic robots, respectively. * *p* < 0.05; ** *p* < 0.01; *** *p* < 0.001.

**Table 3:** Group means and SD of theta ERSPs for high, middle, and low anthropomorphic robots.

Regions of Interest (ROIs)	Time of Interest (TOIs)	H-AR		M-AR		L-AR	
		Mean	SD	Mean	SD	Mean	SD
Frontal cluster	50–380 ms	2.214	0.136	2.229	0.186	1.689	0.151
Parietal-occipital cluster		2.357	0.161	2.759	0.192	2.596	0.248
Frontal cluster	400–1000 ms	0.693	0.100	1.119	0.183	0.699	0.171
Parietal-occipital cluster		0.453	0.154	1.353	0.199	1.087	0.188

## Data Availability

The data used to support the findings of this study are available from the corresponding author upon request.
